# Substitution of White Meat for Red Meat and Diabetes Risk: A Prospective Cohort Study Stratified by Red Meat Intake

**DOI:** 10.3390/nu18040669

**Published:** 2026-02-18

**Authors:** Langrun Wang, Jie Guo, Yiran Guan, Chao Zhang, Ran Wang, Keji Li, Ruixin Zhu, Jingjing He

**Affiliations:** 1 Key Laboratory of Precision Nutrition and Food Quality, Department of Nutrition and Health, China Agricultural University, Beijing 100193, China; b20243311365@cau.edu.cn (L.W.); guojie@cau.edu.cn (J.G.); sy20233313730@cau.edu.cn (Y.G.); zhangchao2023@alu.cau.edu.cn (C.Z.); wangran@cau.edu.cn (R.W.); 2 Department of Nutrition and Food Hygiene, School of Public Health, Peking University, Beijing 100191, China; kejili@bjmu.edu.cn; 3 Research Center for Probiotics, China Agricultural University, Beijing 100193, China

**Keywords:** diabetes, diabetes risk, red meat, white meat, Chinese adults

## Abstract

**Background/Objectives**: Current evidence on the diabetes prevention benefit of substituting red meat with white meat remains inconsistent and is predominantly based on Western populations. This research examined whether the benefits of such dietary substitution depend on habitual red meat intake levels. **Methods**: This prospective analysis included 12,143 adults from the China Health and Nutrition Survey (2004–2015). Dietary intake was assessed by multiple 24 h recalls supplemented by a household food inventory. Incident diabetes was identified via self-report and supplementary biochemical data. Estimates of hazard ratios (HRs) and 95% confidence intervals (CIs) were obtained through Cox proportional hazards modeling. Dose–response relationships were examined using restricted cubic splines. Substitution effects were evaluated within strata defined by baseline red meat intake (<75 vs. ≥75 g/day). **Results**: During 83,046 person-years of follow-up, 687 incident diabetes cases occurred. U-shaped associations were identified for both red meat (lowest risk at 75 g/day) and white meat (lowest risk at 60 g/day) consumption in relation to diabetes risk (*p*-nonlinearity < 0.0001). Substitution was not associated with diabetes risk in the low-intake stratum (<75 g/day, *p* = 0.107). Conversely, in the high-intake stratum (≥75 g/day), replacing 50 g/day of red meat with white meat was associated with a 34% lower diabetes risk (HR: 0.66; 95% CI: 0.51, 0.86; *p* = 0.002). **Conclusions**: Meat intake shows a nonlinear association with diabetes risk in Chinese adults. The potential benefit of substituting white meat for red meat is conditional, with a more pronounced effect observed among individuals with high habitual red meat consumption, which may support the development of targeted dietary guidance for this subgroup.

## 1. Introduction

The escalating prevalence of diabetes poses a substantial burden on global health systems, with China bearing the largest number of adults living with the condition worldwide [1]. In China, the estimated prevalence has risen sharply from less than 7.53% in 2005 to approximately 13.7% in 2023 [2]. This surge coincides with China’s rapid economic development and urbanization, which have precipitated a profound “nutrition transition” from traditional plant-based diets towards dietary patterns richer in refined carbohydrates, edible oils, and, most notably, animal-sourced food [3]. Given that dietary habits represent a key adjustable element affecting the likelihood of developing type 2 diabetes (T2D), identifying specific dietary components that influence risk is crucial for developing effective prevention strategies.

Among dietary factors, the consumption of red meat (primarily pork, beef, and mutton) has been extensively studied in relation to diabetes risk. Substantial evidence from prospective cohorts and meta-analyses, predominantly in Western populations, indicates a positive association between red meat consumption, especially processed varieties, and an increased risk of T2D [4,5,6]. Mechanistically, this association may be mediated through multiple pathways. Compounds naturally present in red meat, such as heme iron and saturated fats, can promote oxidative stress and inflammation [7]. Furthermore, harmful substances formed during cooking (e.g., advanced glycation end products) or generated by gut microbiota from red meat components (e.g., trimethylamine *N*-oxide) have also been implicated in increasing diabetes risk [8,9]. Consequently, many dietary guidelines recommend limiting red meat intake.

Based on these mechanistic considerations regarding red meat, white meat is widely regarded as preferable to red meat. This is primarily due to differences in their nutrient composition. Red meat is a notable dietary source of heme iron and saturated fatty acids (SFAs), which may promote oxidative stress, inflammation, and insulin resistance [10,11,12]. In contrast, white meat generally contains lower levels of these components. Furthermore, fatty fish are a unique source of long-chain omega-3 polyunsaturated fatty acids (PUFAs), such as EPA and DHA, which have anti-inflammatory properties and may beneficially influence glucose metabolism [13,14]. This biological rationale supports the hypothesis that substituting red meat with white meat could be a beneficial dietary strategy for T2D prevention.

However, epidemiological findings regarding the association between dietary white meat and T2D risk remain inconsistent. While some studies suggest a protective effect [15], others report null findings [16,17] or even positive associations [18,19]. Similarly, statistical models estimating the outcomes of replacing red meat with white meat also yield mixed results, with some supporting a beneficial effect [15,20] and others showing no clear benefit [21].

Critically, the current evidence regarding the potential benefit of substituting white meat for red meat on diabetes risk is derived largely from Western populations. Significant differences exist between Eastern and Western populations in the primary types of red meat consumed (e.g., pork-dominant in China vs. beef in the West), consumption levels, culinary practices (e.g., stir-frying/stewing vs. grilling/deep-frying), and genetic susceptibility [22]. Therefore, the generalizability of substitution effects observed in Western settings to the Eastern population is uncertain.

The complexity of this relationship is further underscored by evidence from Eastern populations. A meta-analysis stratified by dietary patterns and geographic regions revealed a notable positive link between red meat and T2D in Western populations, but no significant association in Eastern populations [6]. Moreover, several studies in China and East Asia suggest that the red meat–diabetes relationship may not be linearly positive but rather follow non-linear patterns [23,24]. For example, a 2023 dose–response meta-analysis focusing on East Asian populations reported a U-shaped association between unprocessed red meat and T2D risk [24]. This implies that the health impact of red meat may vary depending on the intake level, with moderate consumption posing minimal risk, whereas higher intake elevates risk substantially. This non-linear pattern raises a pivotal yet under-investigated question: Does the impact of this substitution depend on an individual’s baseline red meat intake? However, previous substitution studies have generally not stratified analyses by baseline red meat intake, limiting the ability to provide nuanced, intake-specific dietary guidance.

To address these knowledge gaps, this prospective study utilizes data from the China Health and Nutrition Survey (CHNS) to: (1) assess the dose–response associations between the consumption of red and white meat and the incidence of diabetes within the Chinese population; and (2) innovatively stratify the population based on habitual red meat intake (low vs. high) and, within each stratum, employ substitution models to quantify and compare the association between replacing red meat with white meat and diabetes risk. We hypothesize that a non-linear relationship exists between red meat consumption and diabetes incidence in Chinese adults, and that the benefit of substituting white meat for red meat will be evident primarily among individuals with habitually high red meat consumption, but not among those with low consumption. This study aims to generate novel, context-specific evidence to inform more targeted dietary recommendations for diabetes prevention in China.

## 2. Materials and Methods

### 2.1. Study Population

This study utilized data from the China Health and Nutrition Survey (CHNS). Employing a multistage, random cluster design, the CHNS recruited a diverse sample from 15 provinces and cities across China since 1989. Specifically, counties within provinces were first stratified by income level, followed by the weighted random selection of counties and major cities (including the provincial capital and a lower-income city). Within these selected areas, villages, townships, and urban/suburban neighborhoods were then randomly chosen as primary sampling units. It is an ongoing public prospective cohort aimed to capture substantial geographic, economic, public resources, and health diversity. Follow-ups are conducted biennially or triennially, with over ten rounds of surveys accomplished thus far. Although not nationally representative, the CHNS constitutes a large-scale cohort for the Chinese population. Its particular strength lies in the detailed dietary assessment combining 3-day, 24 h dietary recalls with household weighing, making it a valuable resource for studying diet–disease relationships in China. The survey protocols were approved by the institutional review boards [25]. All participants provided written informed consent. Detailed cohort characteristics have been described previously [25].

For the present analysis, we designated the 2004 survey wave as the baseline because a different food composition table was used prior to 2004 compared to subsequent surveys, ensuring consistency in nutrient estimation. From the CHNS database, 27,403 participants had available disease history and physical examination data from 2004 onwards. The analytical sample was derived after applying the following exclusion criteria: age < 18 years, pregnant, lactating or disabled, pre-existing diabetes or major chronic diseases (stroke, myocardial infarction, cancer), implausible energy intake (<700 or >5000 kcal/day), and missing key data (such as follow-up information, outcome, dietary, key covariate data. After these exclusions, 12,143 participants were included in the final analytical sample (Figure S1).

### 2.2. Assessment of Outcome

The outcome of interest was incident diabetes. In the CHNS, incident diabetes cases were primarily identified through self-report during the biennial or triennial survey questionnaires. Diabetes ascertainment was based on three self-reported items: (1) confirmation of a doctor’s diagnosis; (2) age of disease onset; and (3) adoption of therapeutic measures, such as medication, lifestyle changes, or traditional practices (qigong) [26]. A participant was considered an incident case if they answered “yes” to question 1, provided a diagnosis age in question 2, or reported any treatment in question 3 in a survey wave subsequent to baseline. In cases of inconsistent reports across waves, the earliest report of diagnosis was used to minimize the impact of recall bias.

To address potential under-reporting, we supplemented self-reported data with available biochemical criteria for a subset of participants. Fasting plasma glucose (FPG) and glycated hemoglobin (HbA1c) were measured only in the 2009 survey. Therefore, for the 2009 data, a fourth criterion (FPG ≥ 7.0 mmol/L or HbA1c ≥ 6.5% [48 mmol/mol]) was additionally applied to define diabetes.

### 2.3. Assessment of Follow-Up Time

Each participant’s initial survey year in which they enrolled in the study and provided complete dietary data served as their baseline. Person-years were determined by the interval between baseline and the earliest of the following: diagnosis of diabetes, final survey before dropout, or the 2015 study cutoff date.

### 2.4. Assessment of Dietary Exposures

Data on dietary consumption were collected via three consecutive 24 h recalls, distributed across two weekdays and one weekend day, combined with a household food inventory weighing method. For cooking oil and condiments, trained interviewers conducted weighing at the household level over a specified period. Individual intake was then estimated based on the individual’s share of household consumption during that period. For all other foods, intake was assessed by trained nutritionists using detailed 24 h recalls over the three days, encompassing all meals, snacks, and beverages. The accuracy of this method has been demonstrated elsewhere, showing a mean relative difference of 1% (74 kcal/day) in total energy intake compared to the gold standard of household food inventory weighing [27].

The primary dietary exposures were intakes of red and white meat. Red meat referred to livestock products (e.g., pork, beef, mutton, and offal). White meat was constituted by poultry and fish. Intakes of total energy, vegetables, and fruits were also adjusted for in the models. All food weights refer to the raw, edible portion. Total energy intake was calculated by matching individual food intake data with the Chinese Food Composition Table and summing the energy contributions from all foods. To address potential confounding from total energy intake and minimize unrelated variability, intakes of red meat, white meat, vegetables, and fruits were energy-adjusted via the residual method after log-transformation, standardized to the mean energy intake of the cohort separately for men and women [28]. To mitigate within-individual variation and approximate long-term diet, a cumulative average intake was computed for each food group using all available dietary surveys from baseline until the event or censoring.

Participants were categorized into quintiles based on their cumulative average consumption of red or white meat for subsequent analyses. For white meat, because the 20th percentile was 0 g/day, all participants with zero intake were assigned to the first category (Q1). The remaining participants were assigned to quartiles corresponding to their positive white meat consumption levels, resulting in a total of five categories.

### 2.5. Assessment of Non-Dietary Covariates

Non-dietary covariates included baseline demographic and lifestyle factors: age (years), sex (men/women), residence (rural/urban), highest educational attainment (categorized as four levels), household income per capita (categorized into four levels based on baseline quartiles: low, medium, high, and very high), body mass index (BMI, kg/m^2^), physical activity level (PAL), smoking status (ever/current vs. never), and alcohol drinking status (yes/no).

Participants underwent anthropometric measurements in the morning after fasting. A calibrated beam balance scale was used to record body weight to the nearest 0.1 kg, while height was measured with a stadiometer accurate to 0.1 cm. Both measurements were taken with participants barefoot and wearing lightweight clothing. From these, BMI was calculated as weight in kilograms divided by the square of height in meters.

PAL was primarily determined based on occupational activity, reported in five categories: very light, light, moderate, heavy, and very heavy. Following the Chinese Dietary Reference Intakes (2000), these categories were converted into multiples of the estimated Basal Metabolic Rate (BMR) for men and women separately (e.g., very light: 1.3 × BMR; light: 1.6 × BMR for men, 1.5 × BMR for women, etc.).

### 2.6. Statistical Analysis

We first explored the dose–response relationships between red meat intake, white meat intake, and diabetes risk using restricted cubic splines (RCS) with three knots (at the 5th, 50th, and 90th percentiles) within Cox proportional hazards models. The SAS macro %RCS_Reg provided by Desquilbet and Mariotti was used [29]. These models were fully adjusted for all dietary and non-dietary variables (mutually adjusted for red and white meat).

Based on the nadir point identified in the RCS analysis for red meat consumption (75 g/day), the total cohort was stratified into two subpopulations for subsequent analyses: Subpopulation 1 (red meat intake < 75 g/day) and Subpopulation 2 (red meat intake ≥ 75 g/day).

Data are reported as mean ± standard deviation, median (interquartile range), or count (percentage) for normal, skewed continuous data, and categorical data, respectively. To evaluate linear trends across meat intake categories, general linear models were applied for continuous variables, and Chi-square trend tests were used for categorical variables; in both cases, the median intake level of red meat in each quintile was entered as an ordinal variable in the model.

Cox proportional hazards regression models were used to estimate hazard ratios (HRs) and 95% confidence intervals (CIs) for the associations of meat intake categories and substitution with diabetes risk. In these models, the dependent variable was incident diabetes (yes/no), with follow-up time (person-years) calculated as detailed in Section 2.3. For the analysis of meat intake categories, red and white meat consumption were entered as quintile-based categorical variables, with the lowest quintile serving as the reference. For the substitution analysis, both meat intakes were modeled as continuous variables. The baseline survey year was used as a stratification variable to account for potential period effects.

Verification of the proportional hazards assumption was performed using interaction terms involving meat intake and log-transformed time; no significant violations were found (all *p* > 0.05). Specifically, in Subpopulation 1 (low red meat intake), the *p*-values were 0.095 for red meat and 0.179 for white meat. In Subpopulation 2 (high red meat intake), the corresponding *p*-values were 0.321 and 0.108, respectively.

For the analysis of red and white meat intake categories, three sequential models were fitted: Model 1 was adjusted for age, gender, BMI, and total energy intake. Model 2 extended Model 1 by further adjusting for all non-dietary factors. Model 3 additionally controlled for energy-adjusted vegetable and fruit intake. Red and white meat intake were mutually adjusted in all models. The lowest intake quintile served as the reference. Statistical significance of linear trends was assessed by modeling the category-specific median intake as a continuous predictor in the regression analyses.

To analyze the substitution effects for red meat, we applied the fully adjusted Cox model (Model 3) including both meat intakes as continuous variables. The HR for the substitution was calculated as exp(β_white − β_red), and its 95% CI was estimated using the standard errors and correlation of these coefficients. This estimates the theoretical change in risk when decreasing red meat by 1 g/day while increasing white meat by the same amount. To present more tangible estimates, we computed the HRs for specific substitution amounts of 30 g/day and 50 g/day.

To evaluate potential effect modification, we conducted stratified analyses based on age (<50 vs. ≥50 years), BMI (<24 vs. ≥24 kg/m^2^), baseline hypertension status, smoking history (ever/current vs. never), and alcohol consumption. Effect modification was formally tested by introducing interaction terms between continuous white meat intake (from the substitution model) and each subgroup variable into the model, with statistical significance assessed via likelihood ratio tests.

All *p*-values were two-sided, and statistical significance was determined by *p* < 0.05. All analyses were performed via IBM SPSS Statistics for Windows, Version 27.0 (IBM Corp., Armonk, NY, USA) and SAS version 9.4 (SAS Institute Inc., Cary, NC, USA).

## 3. Results

The final analytical sample consisted of 12,143 participants (5842 men and 6301 women) recruited between 2004 and 2015. The mean (SD) age at baseline was 45.8 (14.6) years. Over a cumulative follow-up period of 83,046 person-years, 687 new cases of diabetes were recorded. The median cumulative average intakes were 56.1 g/day for red meat and 44.4 g/day for white meat.

**Figure 1:**
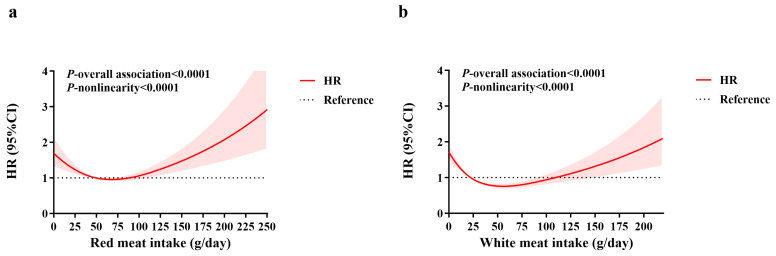
(**a**) Dose–response relationships of dietary red meat intake with diabetes risk; (**b**) Dose–response relationships of dietary white meat intake with diabetes risk. Restricted cubic splines within cox proportional hazards models were used to visualize the dose–response associations, with adjustment for age, gender, BMI, residence area, highest education level, household income level, physical activity level, smoking status, alcohol consumption, history of hypertension at baseline, and dietary intake of total energy, vegetables, and fruits. The reference value was set at the median intake level (corresponding to an HR = 1, indicated by the horizontal solid black line in the figure). The solid red line represents the hazard ratio (HR), the shaded pink area represents the 95% confidence interval (CI) for the estimated HR, while the dashed lines represent the lower and upper bounds of the 95% confidence interval, respectively.

As illustrated in Figure 1a,b, dose–response analyses using restricted cubic splines within Cox proportional hazards models revealed significant U-shaped, non-linear relationships between both red and white meat consumption and diabetes risk (*p*-nonlinearity < 0.0001). Specifically, diabetes risk initially decreased with increasing red/white meat intake at lower levels but increased after intake exceeded specific thresholds. The point of lowest risk was observed at intakes of approximately 75 g/day for red meat (Figure 1a) and 60 g/day for white meat (Figure 1b).

Given this U-shaped relationship, we hypothesized that the health effects of replacing red meat with white meat could vary based on an individual’s habitual red meat consumption. We therefore stratified the cohort into two distinct subpopulations for subsequent analysis, using the nadir of the red meat risk curve (75 g/day) as a cutoff: Subpopulation 1 (lower intake, <75 g/day, n = 8454) and Subpopulation 2 (higher intake, ≥75 g/day, n = 3689).

Baseline characteristics across gender-specific quintiles of red meat intake within each subpopulation are presented in Table 1. In Subpopulation 1 (characterized by lower red meat intake), participants in the upper quintiles of red meat consumption were notably younger, had lower physical activity levels, and had a greater likelihood of residing in urban settings with elevated socioeconomic standing. They also had a lower prevalence of smoking and hypertension but a higher prevalence of alcohol consumption. Variations in diet were also observed, including increased consumption of total energy, white meat, and fruit, alongside reduced vegetable intake (all *p*-values < 0.05). In Subpopulation 2 (higher red meat intake), demographic trends were similar, but key differences emerged: individuals with higher red meat intake had a higher proportion of smokers, and dietary patterns showed lower total energy intake and higher vegetable intake, with no significant differences in alcohol consumption, hypertension, or fruit intake.

The relationships between the consumption of red and white meat and the likelihood of developing diabetes within each subgroup are detailed in Table 2. In Subpopulation 1, both red and white meat intake exhibited significant inverse linear associations with diabetes risk across all models. Under the fully adjusted model 3, a notable inverse correlation was found for red meat intake, where the highest consumption quintile yielded a hazard ratio (HR) of 0.62 (95% CI: 0.46, 0.84; *p*-trend = 0.012) when compared to the lowest quintile. For white meat, the corresponding HR was 0.64 (95% CI: 0.49, 0.85; *p*-trend = 0.001). Additionally, replacing red meat with white meat did not show a significant link to diabetes risk in this subpopulation (*p* = 0.107).

In stark contrast, within Subpopulation 2, red meat intake showed a significant positive linear association with diabetes risk (HR for highest vs. lowest quintile: 1.66; 95% CI: 1.00, 2.75; *p*-trend = 0.009) in the fully adjusted model 3. For white meat, significant protective associations were observed in the second and third quintiles relative to the lowest quintile, but this effect attenuated in higher quintiles, resulting in a non-significant linear trend (*p*-trend = 0.472). Importantly, substitution analysis in this subpopulation revealed that replacing 30 g/day or 50 g/day of red meat with white meat corresponded to a 22% (HR: 0.78; 95% CI: 0.67, 0.91) and 34% (HR: 0.66; 95% CI: 0.51, 0.86) reduction in diabetes risk, respectively (*p* = 0.002 for the overall substitution model).

Subgroup analyses for the meat substitution effect are detailed in Table 3. Within Subpopulation 1, no notable relationships were identified across any of the subgroups categorized by sex, age, BMI, smoking, alcohol drinking, or hypertension status. In Subpopulation 2, the protective effect of substitution appeared more pronounced in men, older adults (≥50 years), individuals with BMI < 24 kg/m^2^, and baseline alcohol drinkers. However, no significant interactions were observed for any of the subgroup variables.

## 4. Discussion

In this prospective cohort investigation involving Chinese adults, we observed significant non-linear, U-shaped relationships between the consumption of both red meat and white meat and the incidence of diabetes. Building upon this fundamental observation, our study further suggested that the health outcomes associated with substituting white meat for red meat may be dependent on an individual’s habitual red meat intake level. Specifically, among participants with a low baseline red meat intake (<75 g/day), higher intake of either meat type correlated with a reduced diabetes risk, and substituting white meat for red meat was not associated with benefit. In stark contrast, among those with a high baseline red meat intake (≥75 g/day), red meat consumption showed a positive linear association with diabetes risk, and a significant reduction in risk was observed when white meat replaced red meat in the diet. These findings provide novel, population-stratified evidence that challenges the notion of a uniform dietary recommendation regarding meat substitution and highlights the potential importance of considering baseline consumption patterns for targeted diabetes prevention.

This study found a U-shaped association for both red and white meat, differing from the linear, positive/negative associations often reported in many Western cohorts [4,30,31,32]. However, this discrepancy is not isolated and finds resonance in emerging research from Asian populations. A recent meta-analysis focusing on East Asia reported a U-shaped association for unprocessed red meat [24], and studies within China have documented similar non-linear patterns for various protein sources [23]. Some studies suggest that very low meat intake in Asian populations might serve as a marker for poverty or poor overall diet quality, whereas moderate intake ensures adequate protein and micronutrient status [33]. The divergence of our findings from some Western studies might be due to the substantially lower range of meat intake in China compared to the West; the “high” intake group in our study (≥75 g/day) might only correspond to a moderate or low-to-moderate intake level in US-based cohorts [34]. It is proposed that the connection between meat consumption and diabetes appears to follow a dose-dependent pattern, where moderate intake as part of a predominantly plant-based diet does not increase risk and could potentially offer health advantages.

The U-shaped association observed in this study, particularly the increased risk at low intake levels and the sharp increase in risk beyond 75 g/day, potentially reflects a balance between physiological nutrient requirements and the cumulative burden of dietary risk factors. Red meat is a rich source of several key nutrients, such as highly bioavailable heme iron, vitamin B12, zinc, and selenium. Moderate consumption might therefore support vital physiological functions, including erythropoiesis, neuroprotection, and antioxidant activity [35,36]. Consequently, it is hypothesized that extremely low intake could lead to a deficiency of these nutrients, potentially disrupting metabolic homeostasis and increasing the risk of dysregulation. On the other hand, when red meat intake exceeds 75 g/day, detrimental mechanisms historically associated with red meat consumption might become dominant, engaging several integrated metabolic pathways. First, as a primary source of heme iron, excessive intake may not only promote oxidative stress via the Fenton reaction [37,38], but also interfere with systemic glucose homeostasis by modulating the adiponectin-hepcidin axis and impairing insulin signaling in hepatic tissues [39]. Second, the high SFA content of red meat can contribute to insulin resistance and inflammation [40]. Specifically, SFAs may act as ligands for Toll-like receptor 4 (TLR4), activating IKKβ/NF-κB and JNK1 signaling cascades that drive the production of pro-inflammatory cytokines (e.g., IL-6, TNF-α), which in turn can induce insulin resistance through inhibitory phosphorylation of insulin receptor substrates [41,42]. Third, red meat, especially if processed or cooked at high temperatures (common in stir-frying or roasting), contains AGEs and HCAs [43], which are thought to interfere with glucose homeostasis and induce systemic inflammation, which are established drivers of T2D. Additionally, aberrant lipid signaling—driven by the accumulation of meat-derived long-chain acyl-CoAs and ceramides—may disrupt mitochondrial β-oxidation and promote lipotoxicity in both skeletal muscle and pancreatic β-cells [44]. Finally, the convergence of these pathways may collectively overwhelm the body’s homeostatic capacity, shifting the risk-benefit balance adversely. The threshold of 75 g/day may thus represent a theoretical point at which this cumulative metabolic burden begins to substantially elevate diabetes risk.

An important observation of our study is the clear effect modification by baseline red meat intake. In the low-intake stratum, where the average consumption was well below the nadir of the risk curve, a rise in consumption of either type of meat correlated with a decreased risk. In this nutritional context, substituting one source of animal protein for another provided no additional advantage, as both appeared beneficial relative to the lowest intake. This may explain why some previous substitution studies, which did not stratify by baseline intake, reported null findings [21]. Conversely, in the high-intake stratum, individuals were already on the upward-sloping, hazardous segment of the red meat risk curve. Here, reducing red meat while increasing white meat intake was linked to a notable decrease in risk—up to 34% for a 50 g/day substitution. Biologically, these observed associations might be explained by the more favorable nutrient profile. Proposed mechanisms include lower levels of potentially harmful components in white meat—such as heme iron (potentially reducing oxidative stress and lipid peroxidation) [10], pro-inflammatory N-glycolylneuraminic acid [45,46], and precursors (carnitine/choline) for gut microbiota-derived trimethylamine N-oxide (TMAO), a compound linked to diabetes risk [47,48], but also from the presence of potentially protective constituents in white meat. These include the relatively higher levels of long-chain n-3 polyunsaturated fatty acids (EPA and DHA) in fish [10], which may possess anti-inflammatory and insulin-sensitizing properties [49] and bioactive peptides in poultry with antioxidant activity [50]. Our stratified results may help reconcile seemingly contradictory literature by suggesting that the benefit of substitution appears conditional becoming more apparent particularly among individuals with high habitual red meat consumption. This pattern may be attributable to the more favorable biochemical profile of white meat, which could potentially help mitigate the metabolic burdens associated with high red meat intake.

Our findings may have important implications for refining dietary guidelines. Current recommendations to limit red meat and replace it with white meat or plant-based proteins are often generalized to entire populations [51,52]. However, our study suggests that a “one-size-fits-all” substitution advice might not be optimal, particularly in populations like China undergoing nutrition transition, where dietary patterns and baseline intakes are highly heterogeneous. For individuals with already low red meat intake, encouraging further reduction or substitution might be unnecessary [53] and could even divert attention from other more critical dietary improvements, such as increasing vegetables, fruit, or whole grain intake [54]. In contrast, public health efforts could prioritize encouraging substitution among those with high red meat consumption, as this group stands to gain the most significant benefit [55]. This nuanced approach may support the development of more personalized and effective dietary strategies for diabetes prevention [56].

The strengths of this study lie in its prospective design, extended long-term follow-up, and the detailed, validated dietary assessment method combining 24 h recalls with household inventory weighing. A key methodological strength is our innovative analytical approach—first identifying a non-linear dose–response relationship, then stratifying the cohort by the empirically derived nadir, and finally applying substitution models within each stratum. This approach directly addresses the conditional nature of dietary effects and moves beyond the assumption of uniform linear associations prevalent in prior research.

Several limitations require cautious interpretation of our findings, notably within the scope of the stratified and substitution analyses. First, while our dietary assessment method is rigorous, self-reported intake is prone to measurement inaccuracies and may not comprehensively reflect individual cooking practices (e.g., precise methods, added fats). Such non-differential misclassification could, however, bias estimates towards the null, suggesting that the true associations, especially within the high-intake stratum, might be stronger. Second, the outcome assessment did not distinguish between types of diabetes (e.g., type 1, type 2, LADA, or MODY). This is an inherent limitation of the CHNS data, in which diabetes was primarily ascertained by self-report. However, given that the vast majority (typically > 90%) of incident diabetes cases in adult populations are T2D, and considering the adult age profile of our cohort (mean age 45.8 years), the observed associations are highly likely to reflect the relationship with T2D. Third, residual confounding remains possible despite extensive adjustment for lifestyle variables. This is particularly relevant for important covariates, such as vegetable intake and physical activity, which were self-reported and thus subject to measurement error. Therefore, we cannot definitively attribute the observed associations solely to meat intake. Notably, information on family history of diabetes was not available in our dataset; this unmeasured factor could confound the associations if individuals with a genetic predisposition adopt different dietary patterns. Fourth, diabetes incidence was primarily ascertained by self-report, potentially leading to under-ascertainment, especially in early or asymptomatic stages. We mitigated this by incorporating biochemical criteria (FPG, HbA1c) for the 2009 survey wave. Fifth, the CHNS cohort, though large and diverse, is not nationally representative, which may affect the generalizability of the absolute intake thresholds (e.g., 75 g/day) to other Chinese subpopulations. Finally, as with all observational studies, causal inference must be made cautiously. The consistent, biologically plausible, and stratified patterns we observed, along with the prospective design, support a likely causal direction for the substitution benefit in high red meat consumers.

## 5. Conclusions

This prospective study indicates that the potential benefit of substituting white meat for red meat on diabetes risk appears to be strictly conditional upon an individual’s habitual red meat intake level. The protective association was significant only among high red meat consumers (≥75 g/day), with no comparable link observed among low-to-moderate consumers. These findings suggest that a recommendation to substitute white for red meat could be considered a targeted preventive strategy for high consumers in China. However, the identified 75 g/day threshold should be applied cautiously and may not be directly generalizable to populations with different dietary patterns. Ultimately, this targeted substitution strategy should be considered as one component of a holistic lifestyle approach to diabetes prevention, which also includes weight management, physical activity, and attention to other psychosocial and metabolic risk factors.

## 6. Future Perspectives

Future research should build upon our key finding that the benefit of meat substitution depends on baseline intake levels. First, short-term randomized controlled trials are needed specifically in individuals with habitual high red meat intake to verify the effects of substitution on their metabolic parameters. Additionally, research integrating deep phenotyping, such as metabolomics, is needed to elucidate the metabolic pathways underlying the observed non-linear associations, ultimately supporting the development of personalized dietary strategies for diabetes prevention in the Chinese population.

## Figures and Tables

**Table 1:** Baseline characteristics of participants across gender-specific red meat intake quintiles within the high and low red meat intake subpopulation *.

	Quintiles of Red Meat Intake					*p* for Trend †
	1	2	3	4	5	
Subpopulation 1 (red meat intake < 75 g/day, n = 8454)						
No. of participants	1690	1690	1692	1690	1692	
Age (y)	47.2 ± 15.6 ‡	46.5 ± 13.9	46.9 ± 14.6	46.8 ± 14.6	45.1 ± 14.4	<0.001
Men (%)	755 (44.7%)	755 (44.7%)	756 (44.7%)	755 (44.7%)	756 (44.7%)	0.997
BMI (kg/m^2^)	23.1 ± 3.3	23.3 ± 3.5	23.3 ± 3.3	23.3 ± 3.5	23.2 ± 3.4	0.637
PAL (×BMR)	1.8 ± 0.3	1.7 ± 0.3	1.7 ± 0.3	1.6 ± 0.3	1.6 ± 0.3	<0.001
Urban residence area, n (%)	331 (19.6%)	390 (23.1%)	525 (31%)	642 (38%)	719 (42.5%)	<0.001
Education level, n (%)						
Low	903 (53.4%)	784 (46.4%)	713 (42.1%)	646 (38.2%)	526 (31.1%)	<0.001
Medium	740 (43.8%)	828 (49%)	874 (51.7%)	917 (54.3%)	1020 (60.3%)	
High	47 (2.8%)	78 (4.6%)	105 (6.2%)	127 (7.5%)	146 (8.6%)	
Household income level, n (%)						<0.001
Low	525 (31.1%)	372 (22%)	281 (16.6%)	232 (13.7%)	220 (13%)	
Medium	433 (25.6%)	360 (21.3%)	338 (20%)	309 (18.3%)	268 (15.8%)	
High	389 (23%)	454 (26.9%)	480 (28.4%)	484 (28.6%)	490 (29%)	
Very high	343 (20.3%)	504 (29.8%)	593 (35%)	665 (39.3%)	714 (42.2%)	
Former or current smoker, n (%)	524 (31%)	551 (32.6%)	513 (30.3%)	494 (29.2%)	467 (27.6%)	0.004
Alcohol consumer, n (%)	480 (28.4%)	502 (29.7%)	592 (35%)	564 (33.4%)	570 (33.7%)	<0.001
Hypertension, n (%)	619 (36.6%)	593 (35.1%)	562 (33.2%)	543 (32.1%)	492 (29.1%)	<0.001
TE (kcal)	2041.8 ± 628	2105.4 ± 576.4	2129.4 ± 572.7	2154.1 ± 564.6	2152.1 ± 566.9	<0.001
Vegetables (g/day)	339.3 ± 221.3	311.9 ± 152.7	317 ± 169.3	317.3 ± 184.9	319.4 ± 175.1	0.014
Red meat (g/day)	0 (0, 3.5)	17.7 (13.6, 21.6)	33.2 (29.3, 37.1)	48.6 (44.9, 52.9)	65.6 (61.5, 70.4)	<0.001
White meat (g/day)	0 (0, 19.6)	11.0 (0, 37.5)	21.6 (0, 53.4)	28.1 (1.2, 55.8)	30.6 (7.4, 59.8)	<0.001
Fruits (g/day)	0 (0, 24.8)	0 (0, 54)	0 (0, 64.2)	12.5 (0, 68.4)	16.9 (0, 75.7)	<0.001
Subpopulation 2 (red meat intake ≥ 75 g/day, n = 3689)						
No. of participants	737	738	738	738	738	
Age (y)	46.0 ± 14.8	44.5 ± 14.2	44.1 ± 13.7	43.4 ± 14.4	43.4 ± 14.5	<0.001
Men (%)	413 (56.0%)	413 (56.0%)	413 (56.0%)	413 (56.0%)	413 (56.0%)	0.979
BMI (kg/m^2^)	23.1 ± 3.3	23.1 ± 3.3	23.2 ± 3.3	22.9 ± 3.2	23.5 ± 3.6	0.123
PAL (×BMR)	1.6 ± 0.3	1.6 ± 0.3	1.6 ± 0.3	1.6 ± 0.3	1.5 ± 0.2	<0.001
Urban residence area, n (%)	349 (47.4%)	368 (49.9%)	385 (52.2%)	403 (54.6%)	458 (62.1%)	<0.001
Education level, n (%)						<0.001
Low	218 (29.6%)	185 (25.1%)	170 (23%)	163 (22.1%)	142 (19.2%)	
Medium	432 (58.6%)	478 (64.8%)	477 (64.6%)	478 (64.8%)	460 (62.3%)	
High	87 (11.8%)	75 (10.2%)	91 (12.3%)	97 (13.1%)	136 (18.4%)	
Household income level, n (%)						<0.001
Low	92 (12.5%)	84 (11.4%)	84 (11.4%)	80 (10.8%)	57 (7.7%)	
Medium	118 (16%)	111 (15%)	90 (12.2%)	85 (11.5%)	59 (8%)	
High	198 (26.9%)	174 (23.6%)	178 (24.1%)	192 (26%)	158 (21.4%)	
Very high	329 (44.6%)	369 (50%)	386 (52.3%)	381 (51.6%)	464 (62.9%)	
Former or current smoker, n (%)	256 (34.7%)	258 (35%)	290 (39.3%)	260 (35.2%)	300 (40.7%)	0.031
Alcohol consumer, n (%)	307 (41.7%)	295 (40%)	268 (36.3%)	300 (40.7%)	332 (45%)	0.199
Hypertension, n (%)	223 (30.3%)	181 (24.5%)	182 (24.7%)	184 (24.9%)	192 (26%)	0.115
TE (kcal)	2157.8 ± 606.6	2166.7 ± 562.2	2168.2 ± 562	2161 ± 622.4	2060.2 ± 671.2	0.004
Vegetables (g/day)	310.3 ± 164.4	324.7 ± 150.3	329 ± 186.7	343.6 ± 232.3	341 ± 236.6	<0.001
Red meat (g/day)	80.2 (77.4, 82.9)	91.8 (88.5, 95)	106.6 (102.5, 111.8)	127.3 (120, 134.6)	171.8 (154.6, 201.8)	<0.001
White meat (g/day)	33.1 (6.8, 62.4)	35.8 (9.6, 66.6)	35.6 (10, 64.1)	35.1 (3.1, 67.6)	38.7 (0, 76.7)	<0.001
Fruits (g/day)	12.6 (0, 65.1)	19.3 (0, 78.8)	15.5 (0, 71.4)	18.2 (0, 75.3)	0 (0, 73.7)	0.530

* Information of non-dietary factors was collected at baseline, and dietary data was estimated as energy-adjusted cumulative average intake from baseline and follow ups. † For the calculation of *p*-values for linear trends, general linear models were applied for continuous variables, and Chi-square trend tests were used for categorical variables; in both cases, the median intake level of red meat in each quintile was entered as an ordinal variable in the model. ‡ mean ± SD, n (%) or p50 (p25, p75) (all such values). Red meat refers to livestock meat, including pork, beef, mutton, and their products (including offal); white meat refers to poultry, fish, and their products. BMI, body mass index; BMR, basal metabolic rate; PAL, physical activity level; TE, total energy; n, number of participants.

**Table 2:** Hazard ratios (95% CI) for diabetes according to red meat and white meat intake, and for the substitution of white meat for red meat, within each red meat intake subpopulation.

	Quintiles of Intake					*p* for Trend
	1	2	3	4	5	
Subpopulation 1 (red meat intake < 75 g/day)						
Red meat						
No. of participants	1690	1690	1692	1690	1692	
Median intake(g/day) *	0.0	17.7	33.2	48.6	65.6	
Cases/person-years	128/11,101	105/12,783	98/12,193	101/12,194	83/12,070	
Model 1	1.00 (Ref.) ‡	0.73 (0.56, 0.95) †	0.66 (0.51, 0.86)	0.68 (0.52, 0.88)	0.61 (0.46, 0.81)	0.004
Model 2	1.00 (Ref.)	0.73 (0.56, 0.95)	0.66 (0.50, 0.86)	0.66 (0.50, 0.87)	0.59 (0.44, 0.80)	0.004
Model 3	1.00 (Ref.)	0.75 (0.57, 0.97)	0.69 (0.52, 0.90)	0.69 (0.52, 0.91)	0.62 (0.46, 0.84)	0.012
White meat						
No. of participants	3142	1327	1328	1328	1329	
Median intake(g/day)	0.0	12.6	28.8	50.3	95.1	
Cases/person-years	282/20,591	54/11,431	49/10,208	57/9804	73/8307	
Model 1	1.00 (Ref.)	0.37 (0.28, 0.50)	0.38 (0.28, 0.52)	0.44 (0.33, 0.58)	0.65 (0.50, 0.85)	<0.0001
Model 2	1.00 (Ref.)	0.37 (0.27, 0.49)	0.37 (0.27, 0.50)	0.42 (0.31, 0.56)	0.60 (0.46, 0.80)	<0.0001
Model 3	1.00 (Ref.)	0.38 (0.29, 0.52)	0.39 (0.29, 0.53)	0.45 (0.33, 0.60)	0.64 (0.49, 0.85)	0.001
White Meat Replacing Red Meat						
Model 3	1 g	1.01 (1.00, 1.01)				0.107
	30 g	1.14 (0.97, 1.35)				
	50 g	1.25 (0.95, 1.65)				
Subpopulation 2 (red meat intake ≥ 75 g/day)						
Red meat						
No. of participants	737	738	738	738	738	
Median intake(g/day)	80.2	91.8	106.6	127.3	171.8	
Cases/person-years	34/4981	25/5013	45/4655	38/4451	30/3605	
Model 1	1.00 (Ref.)	0.89 (0.53, 1.50)	1.73 (1.11, 2.71)	1.79 (1.12, 2.86)	1.76 (1.07, 2.89)	0.003
Model 2	1.00 (Ref.)	0.91 (0.54, 1.52)	1.75 (1.12, 2.75)	1.79 (1.12, 2.85)	1.70 (1.03, 2.80)	0.006
Model 3	1.00 (Ref.)	0.92 (0.54, 1.54)	1.76 (1.12, 2.76)	1.78 (1.11, 2.85)	1.66 (1.00, 2.75)	0.009
White meat						
No. of participants	873	704	704	704	704	
Median intake(g/day)	0.0	17.5	37.8	61.1	111.4	
Cases/person-years	46/4538	34/5098	30/4771	33/4557	29/3741	
Model 1	1.00 (Ref.)	0.58 (0.37, 0.90)	0.53 (0.34, 0.85)	0.66 (0.42, 1.04)	0.71 (0.44, 1.13)	0.43
Model 2	1.00 (Ref.)	0.56 (0.36, 0.88)	0.49 (0.31, 0.79)	0.63 (0.40, 1.00)	0.66 (0.40, 1.07)	0.336
Model 3	1.00 (Ref.)	0.58 (0.37, 0.92)	0.51 (0.32, 0.82)	0.68 (0.43, 1.08)	0.70 (0.43, 1.14)	0.472
White Meat Replacing Red Meat						
Model 3	1 g	0.99 (0.99, 1.00)				0.002
	30 g	0.78 (0.67, 0.91)				
	50 g	0.66 (0.51, 0.86)				

* Intakes were estimated as energy-adjusted cumulative average intake from baseline and follow ups. † Values are HRs (95% CIs) calculated by using Cox proportional hazard analyses. ‡ This denotes the reference category in the model, with its hazard ratio set to 1.00. Model 1: adjusted for age, gender, BMI, dietary intake of TE. Model 2: Model 1 + residence area, highest education level, household income level, PAL, smoking status, alcohol consumption, history of hypertension at baseline. Model 3: Model 2 + dietary intake of vegetables and fruits. Mutual adjustment was performed for dietary red meat and white meat. Red meat refers to livestock meat, including pork, beef, mutton, and their products (including offal); white meat refers to poultry, fish, and their products. Tests for linear trend for HRs were conducted by using the median value for each quintile of intake as a continuous variable. HR, hazard ratio; 95% CI, 95% confidence interval; PAL, physical activity level; TE, total energy.

**Table 3:** Hazard ratios (95% CIs) for the substitution of white meat for red meat in relation to diabetes risk, stratified by selected factors within each red meat intake subpopulation.

	Subgroups	HR and 95% CI of the Analysis of White Meat Replacing Red Meat			*p*	*p*-Interaction †
		1 g/d	30 g/d	50 g/d		
Subpopulation 1 (red meat intake < 75 g/day)	Men (n = 3777)	1.01 (1.00, 1.01) *	1.20 (0.96, 1.51)	1.36 (0.93, 1.98)	0.118	0.422
	Women (n = 4677)	1.00 (1.00, 1.01)	1.08 (0.86, 1.37)	1.14 (0.77, 1.69)	0.514	
	Age < 50 y (n = 4882)	1.00 (0.99, 1.01)	0.92 (0.69, 1.22)	0.86 (0.53, 1.40)	0.545	0.452
	Age ≥ 50 y (n = 3572)	1.01 (1.00, 1.01)	1.26 (1.03, 1.54)	1.47 (1.05, 2.05)	0.026	
	BMI < 24 kg/m^2^ (n = 5235)	1.01 (1.00, 1.02)	1.25 (0.95, 1.64)	1.45 (0.92, 2.28)	0.106	0.626
	BMI ≥ 24 kg/m^2^ (n = 3219)	1.00 (1.00, 1.01)	1.07 (0.87, 1.32)	1.12 (0.79, 1.58)	0.526	
	Non-smoker (n = 5905)	1.00 (1.00, 1.01)	1.09 (0.89, 1.33)	1.15 (0.82, 1.60)	0.423	0.092
	Former or current smoker (n = 2549)	1.01 (1.00, 1.02)	1.23 (0.93, 1.64)	1.42 (0.89, 2.27)	0.146	
	Non-drinker (n = 5746)	1.01 (1.00, 1.01)	1.19 (0.97, 1.47)	1.34 (0.95, 1.89)	0.091	0.731
	Drinker (n = 2708)	1.00 (0.99, 1.01)	1.05 (0.80, 1.38)	1.09 (0.70, 1.71)	0.706	
	Normotensive (n = 5645)	1.00 (0.99, 1.01)	1.07 (0.84, 1.37)	1.12 (0.74, 1.70)	0.587	0.232
	Hypertensive (n = 2809)	1.01 (1.00, 1.01)	1.21 (0.97, 1.50)	1.37 (0.95, 1.97)	0.093	
Subpopulation 2 (red meat intake ≥ 75 g/day)	Men (n = 2065)	0.99 (0.99, 1.00)	0.78 (0.65, 0.95)	0.67 (0.48, 0.92)	0.013	0.993
	Women (n = 1324)	0.99 (0.98, 1.00)	0.80 (0.60, 1.07)	0.69 (0.43, 1.11)	0.127	
	Age < 50 y (n = 2365)	0.99 (0.99, 1.00)	0.80 (0.64, 1.01)	0.69 (0.47, 1.02)	0.063	0.725
	Age ≥ 50 y (n = 1324)	0.99 (0.98, 1.00)	0.76 (0.61, 0.94)	0.63 (0.44, 0.91)	0.012	
	BMI < 24 kg/m^2^ (n = 2300)	0.99 (0.98, 0.99)	0.63 (0.48, 0.83)	0.46 (0.29, 0.74)	0.001	0.409
	BMI ≥ 24 kg/m^2^ (n = 1389)	1.00 (0.99, 1.00)	0.87 (0.72, 1.05)	0.79 (0.58, 1.09)	0.152	
	Non-smoker (n = 2325)	0.99 (0.98, 1.00)	0.77 (0.62, 0.96)	0.65 (0.45, 0.94)	0.021	0.911
	Former or current smoker (n = 1364)	0.99 (0.99, 1.00)	0.79 (0.63, 0.99)	0.68 (0.47, 0.99)	0.044	
	Non-drinker (n = 2187)	0.99 (0.99, 1.00)	0.81 (0.64, 1.01)	0.70 (0.48, 1.02)	0.062	0.816
	Drinker (n = 1502)	0.99 (0.98, 1.00)	0.77 (0.62, 0.97)	0.65 (0.45, 0.94)	0.023	
	Normotensive (n = 2727)	1.00 (1.00, 1.01)	0.75 (0.59, 0.95)	0.62 (0.41, 0.92)	0.295	0.295
	Hypertensive (n = 962)	0.99 (0.99, 1.00)	0.82 (0.66, 1.01)	0.71 (0.50, 1.01)	0.056	

* Values are HRs (95% CIs) calculated by using Cox proportional hazard analyses in Model 3 adjusted for age, gender, BMI, residence area, highest education level, household income level, PAL, smoking status, alcohol consumption, history of hypertension, dietary intake of TE, vegetables and fruits. Mutual adjustment was performed for dietary red meat and white meat. † The *p* value for the interaction term was obtained by including the product term of white meat intake and the subgroup variable in the model. Red meat refers to livestock meat, including pork, beef, mutton, and their products (including offal); white meat refers to poultry, fish, and their products. BMI, body mass index; HR, Hazard ratio; 95% CI, 95% confidence interval; PAL, physical activity level; TE, total energy; n, number of participants; y, year.

## Data Availability

The data presented in this study are openly available in [https://chns.cpc.unc.edu/data/datasets/] (accessed on 1 November 2025).

## Supplementary Materials

The following supporting information can be downloaded at: https://www.mdpi.com/article/10.3390/nu18040669/s1, Figure S1. Participants’ flow chart.
