# Supplementary figures and images for: Substitution of White Meat for Red Meat and Diabetes Risk: A Prospective Cohort Study Stratified by Red Meat Intake
Source: Nutrients. 2026 Feb 18;18(4):669. doi: 10.3390/nu18040669 (PMC12942914; doi:10.3390/nu18040669)

## Supplementary materials

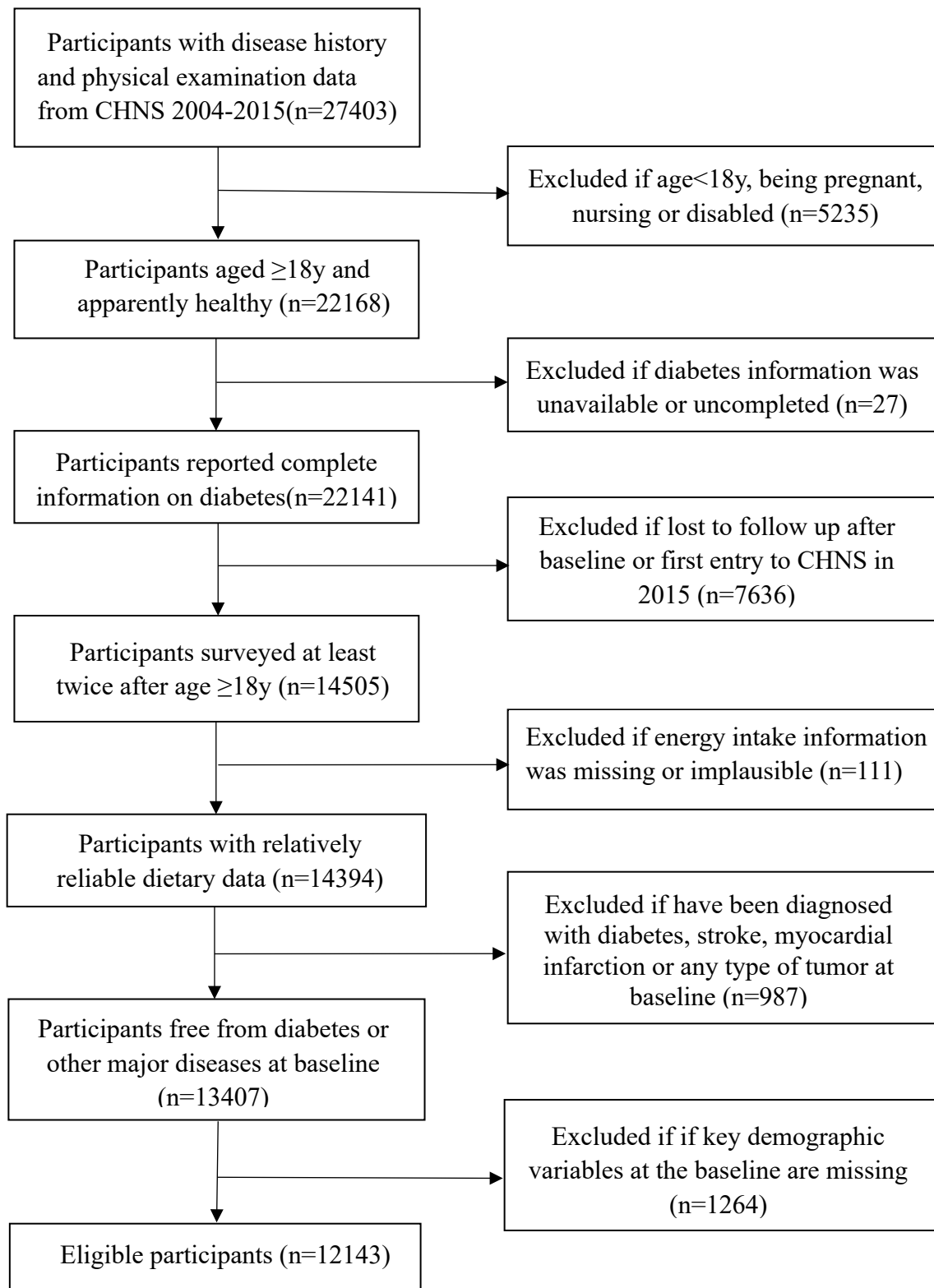

**Figure S1. Participants' flow chart**

Supplement: Supplementary file 1 [file nutrients-18-00669-s001.zip › nutrients-4120521-supplementary.pdf]
